# Treatment of Central Neurocytoma

**DOI:** 10.3390/cancers17122005

**Published:** 2025-06-16

**Authors:** Anna Michel, Jan Rodemerk, Laurèl Rauschenbach, Pikria Ketelauri, Oleh Danylyak, Ramazan Jabbarli, Philipp Dammann, Anne-Kathrin Uerschels, Marvin Darkwah Oppong, Oliver Gembruch, Yahya Ahmadipour, Andreas Junker, Ulrich Sure, Karsten Henning Wrede

**Affiliations:** 1 Department of Neurosurgery and Spine Surgery, University Hospital Essen, University Duisburg-Essen, Hufelandstraße 55, 45147 Essen, Germany; jan.rodemerk@uk-essen.de (J.R.); laurel.rauschenbach@uk-essen.de (L.R.); pikria.ketelauri@uk-essen.de (P.K.); oleh.danylyak@uk-essen.de (O.D.); ramazan.jabbarli@uk-essen.de (R.J.); philipp.dammann@uk-essen.de (P.D.); ann-kathrin.uerschels@uk-essen.de (A.-K.U.); marvin.darkwahoppong@uk-essen.de (M.D.O.); oliver.gembruch@uk-essen.de (O.G.); yahya.ahmadipour@uk-essen.de (Y.A.); ulrich.sure@uk-essen.de (U.S.); karsten.wrede@uk-essen.de (K.H.W.); 2 Center for Translational Neuro- & Behavioral Sciences (C-TNBS), University Duisburg Essen, 45147 Essen, Germany; andreas.junker@uk-essen.de; 3 German Cancer Consortium (DKTK) Partner Site, University Hospital Essen, 45147 Essen, Germany; 4 German Cancer Research Center (DKFZ) Division Translational Neurooncology at the West German Cancer Center (WTZ), DKTK Partner Site, University Hospital Essen, 45147 Essen, Germany; 5 Department of Neuropathology, University Hospital Essen, University Essen, 45147 Essen, Germany

**Keywords:** central neurocytoma, atypical central neurocytoma, pseudorosette, adjuvant radiotherapy

## Abstract

**Objective:** Central neurocytomas (CNs), classified as CNS (central nervous system) grade 2 tumors, are exceptionally rare tumors, accounting for approximately 0.1–0.5% of all intracranial neoplasms, and are typically characterized by a benign clinical course and frequent association with hydrocephalus. This study aims to present a comprehensive analysis of surgical and adjuvant therapies for CN. **Methods:** The study comprised all patients who underwent microsurgical tumor removal in our center over the past decade (2013–2023). Clinical manifestations, surgical and adjuvant therapy approaches, MRI and histological findings, clinical outcomes, and recurrence-free survival were evaluated. **Results:** A total of eleven patients (six men, mean age of 28.0 years; five women, mean age of 53.6 years) underwent surgical treatment. Intraventricular tumors were the most common (72.7%, n = 8). The predominant presenting symptoms were headache and visual disturbances. All tumors exhibited contrast enhancement on MRI. Hydrocephalus was present in five patients. The Ki67 proliferation index ranged from 2% to 10%, with nine patients exhibiting Ki67 > 3%. The median recurrence-free survival was 38.0 months (IQR: 25.0–53.0). The most severe postoperative complications included aphasia, hemiparesis, and memory impairment, resulting in a postoperative Karnofsky Performance Status (KPS) below 70% in five patients. Follow-up assessments showed significant symptomatic improvement in all affected patients. **Conclusions:** Gross total resection is the recommended first-line therapy with favorable neurological outcomes and for atypical CN as well. Adjuvant radiotherapy should be reserved for tumor progression and recurrence. The role of adjuvant chemotherapy remains unclear, but it may be an option for CN with a high proliferation index.

## Simple Summary

The rare tumor entities called central neurocytomas (CNs) are classified as grade 2 CNS (central nervous system) tumors and are characterized by a benign clinical course. All patients who underwent surgery at our center in the last decade (2013–2023) were included in this study. Of the eleven patients (six men, mean age of 28.0 years; five women, mean age of 53.6 years), eight were localized intraventricularly, and the three others were in the pineal region, corpus callosum, and parietal cortex. Headache and visual disturbances were the main symptoms, while five patients presented with hydrocephalus. In total, nine patients had a Ki67 value of >3%. The median recurrence-free survival was 38.0 (IQR: 25.0–53.0) months. Despite some of the most serious postoperative complications, all affected patients showed a significant improvement in symptoms with a KPS ≥ 70%. Complete resection should be the treatment of choice, and adjuvant radiotherapy should be considered for recurrence and progression.

## 1. Introduction

Central neurocytomas (CNs) are rare brain tumors accounting for 0.1–0.5% of all primary intracranial tumor incidences and are predominantly located intraventricularly [1,2]. Initially described in 1982, these tumors typically affect young adults and display distinct histological and immunohistochemical features [3]. The World Health Organization (WHO) has classified this entity as a grade 2 tumor since 2021 [4]. The majority of CN are characterized by a biphasic pattern with clusters of small round cells resembling neuronal precursors, often forming a perivascular pseudorosette arrangement. Immunohistochemical markers, such as synaptophysin and neuron-specific enolase, aid in confirming the neuronal origin of these tumors [5,6,7]. In the literature, some cases of atypical CN were described and characterized with a high Ki67 index and high potential of recurrence [8].

Radiologically, CNs typically present as well-circumscribed, contrast-enhancing lesions, but some cases also show diffuse contrast enhancement [9,10]. Calcifications are commonly detected in these tumors on both CT (computed tomography) and MRI (magnetic resonance imaging), and their characteristic intraventricular location frequently leads to obstructive hydrocephalus [11]. Advances in imaging modalities, such as MRI and CT, have improved the preoperative characterization of CN [12]. However, differentiating CN from other intraventricular tumors like oligodendrogliomas and ependymomas remains challenging due to overlapping imaging features. As a result, tissue-based immunohistochemistry remains the gold standard for establishing a definitive diagnosis [9,13].

Surgical resection remains the primary treatment for CNs, with the goal of achieving maximally safe tumor removal to relieve symptoms and reduce the risk of recurrence [14]. Despite their generally benign nature, the potential for aggressive progression and recurrence necessitates thorough discussion of adjuvant therapies, including radiotherapy. In fact, studies confirm radiotherapy as an option for recurrent or incompletely resected CN, as well as for CN with a higher Ki-67 index [15,16,17]. Current studies on systemic treatment options have highlighted the potential efficacy of temozolomide in CN with elevated Ki-67 levels [18].

CNs are a rare and complex challenge in neuro-oncology, owing to their potential for atypical behavior and recurrence despite a generally benign histology. These characteristics highlight the importance of a multidisciplinary treatment approach. Due to the low number of cases and the occasionally heterogeneous behavior of these tumors, standardized and evidence-based treatment algorithms are still lacking. Therefore, the publication of case series is essential to expand our knowledge on CNs and to enable analysis within the framework of systematic meta-analyses, particularly through studies that report on treatment outcomes and long-term follow-ups. Our study aims to contribute to this growing body of knowledge.

## 2. Materials and Methods

This study was performed in accordance with the Declaration of Helsinki and was approved by the local ethics committee of the University Hospital Essen (local register number 24-12100-BO).

### 2.1. Patients’ Cohort

All patients aged over 16 years with CN (CNS WHO grade 2) who underwent surgical treatment at our institution between January 2013 and December 2023 were included in this analysis (Table 1). Tumors were graded according to the WHO Classification of CNS Tumors (2016 or 2021 editions). Each case was reviewed preoperatively by our neuro-oncology tumor board, and surgical indications were determined through an interdisciplinary decision-making process. The choice of adjuvant therapy was tailored to the individual characteristics of each patient and determined as part of an interdisciplinary discussion at our neuro-oncology conference. CNS is classified as a WHO grade 2 CNS tumor, with some cases having a Ki67 index of over 3–5%. Considering the risk of recurrence, adjuvant radiotherapy is still worth considering. Retrospective data collection from electronic medical records included patient age and gender; radiological findings, such as MRI, CT, and PET-CT; follow-up information, including MRI and clinical status; and histopathological diagnosis. Clinical and neurological status before and after surgery was assessed using the Karnofsky Performance Status (KPS) scale.

### 2.2. Data Analysis

Statistical analyses were conducted with SPSS software version 29 (IBM, Chicago, IL, USA), and results are reported as mean values ± standard deviations. Follow-up time variables were presented as median values with interquartile ranges (IQR, 25–75%) or as the number of cases (with percentages), as appropriate. Kaplan–Meier analysis was used to assess overall survival and progression-free survival. Recurrence-free survival was defined as the interval between surgery and radiologically confirmed recurrences on MRI. T1-, T2-, and contrast-enhanced images of the preoperative MRI scans were analyzed to evaluate the radiological parameters of the CN. Statistical significance was set at *p* < 0.05.

## 3. Results

### 3.1. Cohort’s Characteristics

**Figure 1:**
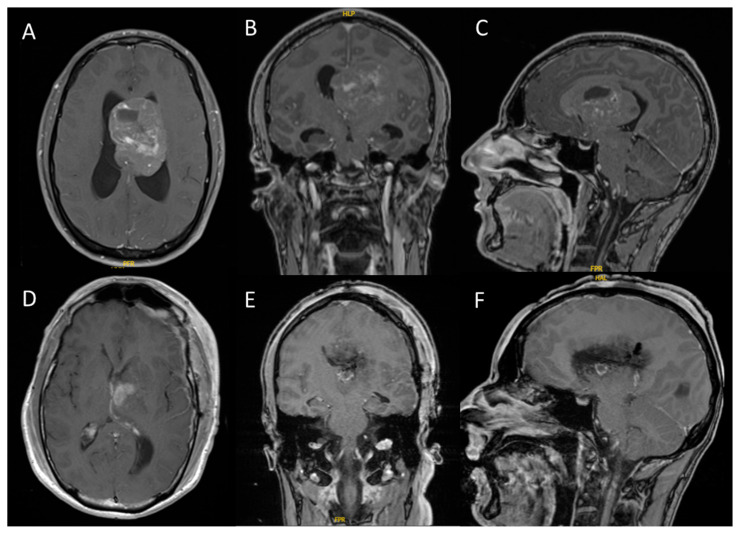
Intraventricular CN with hydrocephalus ((**A**–**C**): preoperative T1-weighted MRI with gadolinium contrast; (**D**–**F**): postoperative T1-weighted MRI with gadolinium contrast). These representative images are from case 1, a 31-year-old male who presented with headaches and cognitive deficits over the previous eight weeks. All tumors demonstrated contrast enhancement on MRI, with some cases exhibiting diffuse enhancement.

From January 2013 to December 2023, a total of 11 patients aged between 16 and 73 years with CN were treated at our center. The majority of tumors were located intraventricularly (n = 8), while the remaining three cases were situated in the pineal region, parietal lobe, and adjacent to the corpus callosum. Following the contrast-enhanced MRI evaluation (see Figure 1), gross total resection was achieved in eight cases, while the remaining three patients underwent subtotal resection. All microsurgical tumor resections were performed using either a transcallosal or transcortical approach with continuous electrophysiological monitoring and neuronavigation. Temporary external ventricular drainage was required in seven patients, and three patients ultimately required permanent ventricular shunting due to persistent hydrocephalus.

### 3.2. Cases 1–8: Intraventricular CN

In summary, five male patients (mean age: 24.4 ± 6.47 years) and three female patients (mean age: 47.7 ± 27.79 years) presented with intraventricular CNS disease (mean age: 33.1 ± 19.74 years). At the time of diagnosis, the most common presenting symptoms included headaches (n = 5), visual disturbances (n = 4), and cognitive impairments (n = 3). All patients had a preoperative KPS greater than 70%. Half of all patients had an initial postoperative KPS of less than 70%. However, all patients recovered well over time, and their symptoms regressed, resulting in a KPS of over 70% in all patients in the long-term follow-up. The median follow-up was 50.0 months (IQR: 28.0–111.0). A total of five patients with intraventricular CN were temporarily treated with external ventricular drainage (EVD) during primary surgical therapy. During the inpatient stay, three patients required permanent CSF drainage; therefore, a VP (ventricular–peritoneal) shunt was inserted in 33.3% of the intraventricular CN cases. We detected four cases of tumor recurrence in follow-up controls. The median recurrence-free survival was 33.0 (IQR: 24.0–89.75, range: 19–158 months). GTR was performed in six cases. Three patients underwent adjuvant radiotherapy despite the absence of residual tumors, based on the neuro-oncology conference’s recommendation. All cases showed a Ki67 index greater than 3% (median: 5.0% [IQR: 4.0–5.0])

#### 3.2.1. Case 9: CN in the Pineal Region

A 73-year-old female patient presented with a one-year history of gait disturbance. An MRI revealed a contrast-enhanced tumor in the pineal region. Due to postoperative bleeding, the patient underwent re-operation and required a VP shunt for hydrocephalus. Complete resection was achieved. Follow-up examinations showed no recurrence, and the patient recovered successfully from postoperative ocular motility disorders.

#### 3.2.2. Case 10: CN in Corpus Callosum

A 52-year-old female patient presented with vertigo symptoms that had occurred for several weeks. An unclear mass was found in her corpus callosum. This mass could be completely resected. The Ki67 index was greater than 10%. Adjuvant radiotherapy was performed. There was no recurrence during follow-up examinations up to four years, and there was no neurological deficit.

#### 3.2.3. Case 11: CN in Parietal Cortex

A 46-year-old male patient presented with a few days of visual disturbance and a focal seizure. He was diagnosed with a parieto-occipital lesion. He underwent partial resection and adjuvant radiotherapy. During the nearly four-year follow-up period, there was no recurrence, and the patient was symptom-free.

### 3.3. Histopathological and Immunohistochemical Features

**Figure 2:**
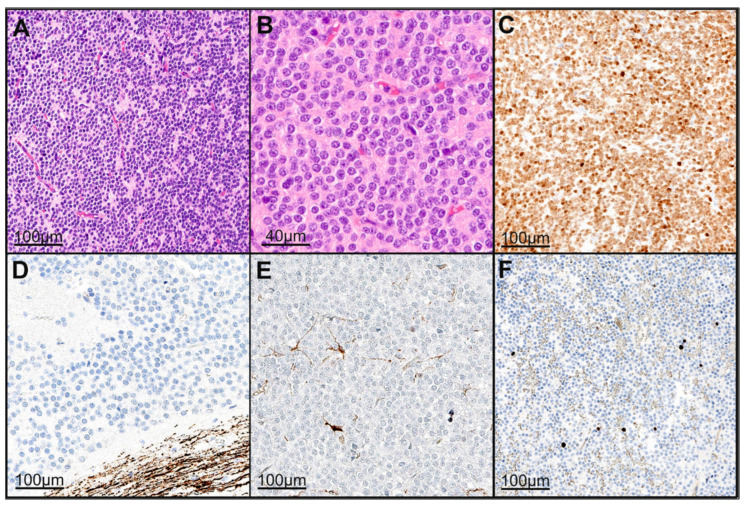
Histopathological analysis revealed a neuroectodermal tumor with a moderate to slightly increased cellularity on HE staining (**A**,**B**). The tumor cells were positive for NeuN (**C**). Neurofilament staining highlighted only the surrounding brain tissue, clearly delineating the tumor from adjacent tissue (**D**). GFAP staining showed positive labeling in only a few infiltrated resident astrocytes (**E**). The Ki-67 proliferation index was approximately 1% (**F**). The histopathologic presentation was based on the specimens of case 5. Abbreviations: HE: hematoxylin–eosin staining, GFAP: glial fibrillary acidic protein, NeuN: Hexaribonucleotide Binding Protein-3, Ki-67: marker of proliferation.

In all cases, the histopathological diagnosis was CN, CNS WHO grade II. HE (hematoxylin–eosin) staining characterized the morphology of tumor tissue. A biphasic pattern was observed, with clusters of small round cells resembling neuronal precursors that often formed a perivascular pseudorosette arrangement. The proliferation index ranged from 2 to 10%. Nine patients showed a Ki67 index greater than 3% and were classified as atypical. The distribution of the Ki67 values appeared as follows: Ki67 = 2%, n = 2; Ki67 = 4%, n = 2; Ki67 = 5%, n = 5; and Ki67 = 10%, n = 2. An increased mitotic rate was observed in two patients (1–2/10 HPF (high-power field)). Overall, immunohistochemical markers such as synaptophysin (positive staining in nine cases) and GFAP (glial fibrillary acidic protein) (positive staining in three cases) were specifically identified in our cases. The entire cohort tested negative for P53 (tumor protein P53) and EMA (epithelial membrane antigen). Five patients showed positive staining for NeuN (Hexaribonucleotide Binding Protein-3) (see Figure 2).

### 3.4. Adjuvant Treatment

Adjuvant radiotherapy was administered to seven patients. Among these, two patients who underwent partial resection received a total radiation dose of 54–60 Gy. Four patients received the same dose range following gross total resection. In addition to conventional stereotactic radiotherapy, two patients were treated with proton therapy. One patient did not receive radiotherapy after initial gross total resection but was treated with 54 Gy of proton therapy upon recurrence. No patients in this cohort received adjuvant treatment with temozolomide or other systemic therapies.

### 3.5. Follow-Up, Overall Survival, and Recurrence-Free Survival

**Figure 3:**
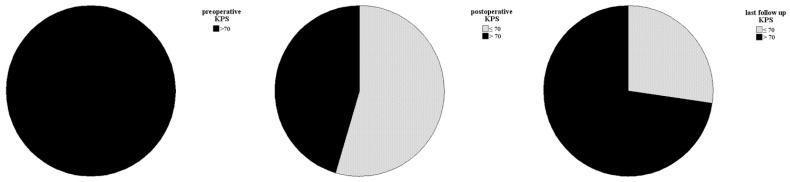
Clinical and neurological statuses were assessed preoperatively, postoperatively, and during follow-up. Follow-up data showed a significant improvement in symptoms for all patients, with a KPS of 70% or higher.

**Figure 4:**
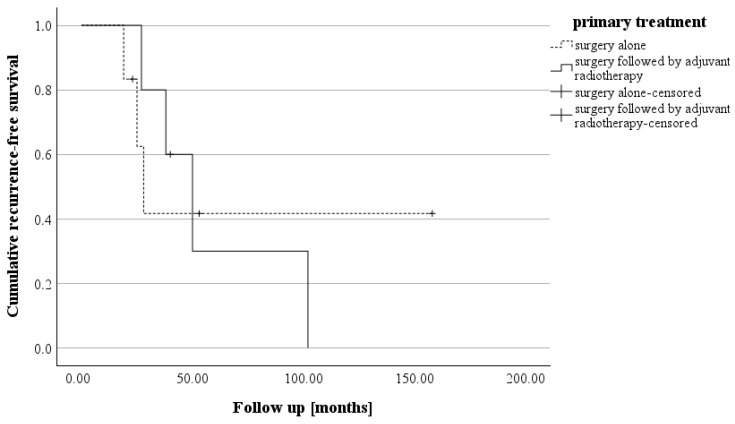
The Kaplan–Meier curve illustrates recurrence-free survival in the two groups, i.e., those treated with surgery alone (median: 26.5 months) and those who received surgery followed by adjuvant radiotherapy (median: 40.0 months). (log rank: *p* = 0.922).

**Figure 5:**
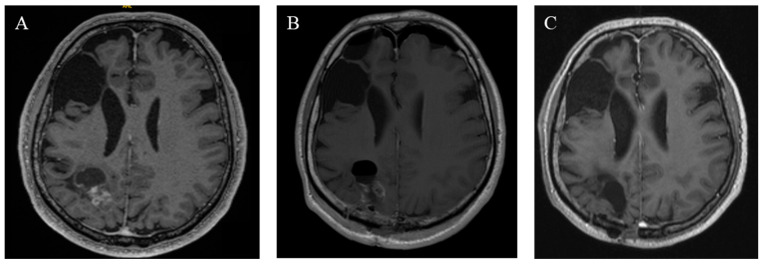
Radiographic findings of case 11. (**A**) Preoperative T1 MRI with a contrast agent. (**B**) Postoperative MRI on the first postoperative day. (**C**) Three years of follow-up MRT after adjuvant radiation.

The initial follow-up was conducted three to six months after surgery. Over the past 10 years, there have been no deaths among patients with CN who were treated at our center. A postoperative assessment of KPS revealed that four patients had a KPS below 70%. However, subsequent evaluations demonstrated significant clinical and neurological improvements. All patients (n = 11) achieved a KPS of 70% or higher, with only three patients having a KPS exactly at 70% (see Figure 3). The patients who suffered a recurrence were treated individually. One patient received adjuvant radiotherapy (case 11). In case 9, a new microsurgical resection was performed for recurrence. Follow-up examinations were maintained in the cohort at the same 6-month intervals. The median recurrence-free survival of the cohort was 38.0 months (IQR: 25.0–53.0). Analysis of recurrence-free survival showed no significant difference based on adjuvant therapy. Patients who underwent surgery alone had a recurrence-free survival of 26.5 months (IQR: 22.0–79.25), while those who received adjuvant radiotherapy after surgery had a recurrence-free survival of 40.0 months (IQR: 32.5–76.0) (see Figure 4). The morphological characteristics of all 11 are shown in Figure 5, as a preoperative MRI, postoperatively and after adjuvant radiotherapy.

## 4. Discussion

CN is a very rare entity of intracranial tumors. This study analyzed eleven cases, with nine classified as atypical CN. The focus of this study was on surgical and adjuvant treatment strategies and the evaluation of clinical and histopathological characteristics in relation to recurrence-free survival.

### 4.1. Roles Adjuvant Radiotherapy and Adjuvant Systemic Therapy

Gross total resection is considered the preferred initial treatment [15,19]. Adjuvant radiotherapy remains a subject of debate, with some studies advising against its use following GTR [17,20]. Adjuvant radiotherapy is more commonly recommended in cases of recurrent CN (tumors with a high proliferation index) or after PTR [15,16,19,21]. A standardized guideline for adjuvant radiotherapy in CN has not yet been established, mainly due to the rarity of this tumor and the lack of high-volume data. In 2024, González et al. reported a rare case of atypical CN with a Ki67 index of 7%, suggesting that the Ki67 index should be incorporated into future analyses and treatment decision-making. The prognostic significance of the Ki67-labeling index is supported by multiple studies, which have shown that higher Ki67 values are associated with increased risk of recurrence and a more aggressive clinical course [8]. Previous studies have demonstrated that an elevated Ki67 index has significant prognostic value, with patients diagnosed with atypical CN exhibiting a higher incidence of local recurrence [22,23]. Several large cohort studies have demonstrated the importance of a 3% Ki67 cutoff, as well as a higher recurrence rate for CN with a Ki67 ≥ 3%. These studies have also shown the importance of GTR for longer progressive-free survival in these cases [6,14]. On the other hand, adjuvant radiation therapy could improve recurrence-free survival in both typical and atypical CNs [19]. Another important consideration is the presence of CN in pediatric patients. Of the cases in this cohort, two involved adolescents, i.e., a 16-year-old female (case 6) and a 17-year-old male (case 7). Both of these patients exhibited elevated proliferation indices. Case 6 underwent gross total resection; however, tumor progression was noted after two years, prompting radiotherapy. Case 7 was managed with a subtotal resection followed by adjuvant radiotherapy. Neither patient experienced recurrence after 23 and 40 months of follow-up, respectively. These cases support the recommendations of other studies, which state that radiotherapy should be considered for pediatric patients, especially in cases of recurrence, partial resection, or increased proliferation rates [24,25,26]. The risks associated with irradiation, including potential delayed complications such as radiation necrosis and leukoencephalopathy, must be carefully weighed; therefore, stereotactic irradiation should be reserved for selected cases. The optimal radiation dose for both adults and children with CN has not been precisely established. However, several studies have proposed that a dose of at least 54 Gy may be appropriate, with some recommending 56–60 Gy in cases of atypical CN or after subtotal resection [27]. A comprehensive therapeutic strategy has yet to be established, but both our experiences and the literature suggest that this approach may be a viable treatment option for selected individual cases. In addition to adjuvant radiotherapy, the use of adjuvant temozolomide is also being considered. Temozolomide is primarily indicated for high-grade, aggressive tumors such as glioblastomas [28]. This is not the case for typical neurocytomas. Furthermore, there is not enough evidence in studies that support its efficacy. However, case reports of atypical central neurocytomas suggest a possible prognostic benefit. This option should be taken into account when managing more aggressive or recurrent CNs. The first findings supporting this approach were published by Patel et al. in 2019 [18]. We have not utilized temozolomide therapy in our cohort. At this time, it remains uncertain whether a general recommendation can be made for adjuvant combined radiotherapy and temozolomide in cases of atypical and recurrent CNs.

### 4.2. CN in the Fourth Ventricle

Only seven cases of CN located in the fourth ventricle have been previously reported in the literature [29,30,31,32,33,34,35]. Of these, four exhibited an increased proliferation index, indicating features of atypia. In our own cohort, we also identified one case (case 2) with CN in this rare fourth ventricle location, which demonstrated a Ki67 index greater than 4%. The patient was 68 years old. Notably, the patients in these reported cases tend to be older than those in the typical CN cohort [35]. Our findings further support the observation that atypical features are more frequently seen in CN arising in this uncommon location. In our case, GFAP positivity was confirmed upon histopathological examination. The literature suggests a higher recurrence rate for CN in the fourth ventricle [36]. However, since CN is generally known for its potential for late recurrence, long-term clinical and radiological follow-ups are especially important for patients with tumors in this location.

### 4.3. Long-Term Follow-Up

CN is known to have a risk of recurrence, which can sometimes occur many years after the initial surgery. Long-term studies have documented cases where recurrence or even tumor dissemination appeared more than a decade postoperatively [37]. This underscores the importance of extended follow-ups to ensure timely detection and management of late recurrences.

## 5. Limitations

The retrospective nature of this study and the small cohort size are acknowledged limitations. Nevertheless, the therapies analyzed here are crucial for informing future research on this rare entity and may offer valuable insights into treatment strategies for CN. To better understand long-term clinical outcomes and the role of adjuvant therapies, larger studies with extended follow-up periods are needed. Despite these limitations, our study presents a relatively large cohort of atypical CN cases, and our findings may help guide future investigations in this field.

## 6. Conclusions

Whenever possible, complete tumor resection should be pursued. There are currently no standardized recommendations for adjuvant therapy after the (partial) resection of CN. In our cohort, adjuvant radiotherapy did not show a clear benefit over observation alone. Given the tumor’s CNS WHO grade 2 classification and generally favorable surgical outcomes, routine radiotherapy is not advised, and its potential side effects should be considered. Long-term MRI and clinical follow-ups are essential to ensure timely detection and management of late recurrences. Larger studies are needed to clarify the role of adjuvant systemic therapies, especially for CN with a high Ki67 index.

## Figures and Tables

**Table 1:** Overview of demographic and clinical characteristics of patients diagnosed with CN, CNS WHO grade 2.

Case Nr	Gender	Age [Years]	Follow Up After OP [Months]	Status First Follow Up **	rec. Free Survival	Adjuvant Radiatio	Preop. KPS	Postop KPS	Last Follow Up KPS	Location	Status of Resection	Surgical Approach	EVD	VP Shunt	TI of Initial Symptoms	Headache	Focal Epilepsy	Visual Disturbance	Memory/Concentration Disorder	Postop. Complication/ Symptoms
1	m	31	19	no rec.	19	no	100%	50%	70%	IV	GTR	transcortical	yes	yes	8 weeks	yes	no	yes	yes	Memory disorder, aphasia, hemiparesis right arm accentuated
2	f	68	28	no rec.	28	no	90%	80%	90%	IV	GTR	transcallosal	yes	no	7 months	no	no	no	no	none
3	m	18	190	rec.	158	no	90%	30%	70%	IV	GTR	transcallosal	no	yes	2 weeks	yes	yes	yes	no	Postoperative bleeding, spastic hemiparesis on the right, temporary tracheostomy
4	f	59	55	no rec.	27	60 Gy	100%	90%	90%	IV	PTR	transcallosal	no	no	2 years	no	yes	no	yes	none
5	m	29	38	no rec.	38	59.4 Gy	100%	50%	70%	IV	PTR	transcallosal	yes	no	2 weeks	no	no	yes	yes	Meningitis, global aphasia
6	f	16	111	no rec.	102	60 Gy	90%	70%	80%	IV	GTR	transcallosal	no	no	unclear	yes	no	no	no	Hemiparesis on the right, tremor of the hands
7	m	17	110	Tr	23	no	100%	60%	80%	IV	PTR	transcallosal	yes	yes	1 week	yes	no	yes	no	Hemiparesis right
8	m	27	113	Tr	53	no	100%	80%	100%	IV	PTR	transcallosal	yes	no	unclear	yes	no	no	no	none
9	f	73	25	no rec.	25	no	80%	40%	80%	PN	GTR	transcallosal	yes	yes	1 year	no	no	no	no	postoperative bleeding, vertical gaze palsy and a right-sided bulbar motor disorder.
10	f	52	50	no rec.	50	59.4 Gy	80%	80%	90%	CC	GTR	transcallosal	no	no	2 weeks	no	no	no	no	none
11	m	46	40	rec.	40	60 Gy *	80%	80%	80%	PO	GTR	transcortical	yes	no	some days	no	yes	yes	no	hemianopsia

Abbreviations: Nr.: number; m: male; f: female; preop.: preoperative; postop.: postoperative; KPS: Karnofsky Performance Status scale; PTR: partial tumor resection; GTR: gross total resection; adj.: adjuvant; RT: radiotherapy; EVD: external ventricular drainage; VP: ventriculo-peritoneal shunt; rec.: recurrence; IV: intraventricular; CC: corpus callosum; PN: pinealis loge; PO: parieto-occipital; TI: time interval; *: adj. radiation after rec.; OP: operation; Tr: tumor residue; **: follow-up after 6 months.

## Data Availability

The data presented in this study are available upon request from the corresponding author. The data are not publicly available due to ethical restrictions.

## Abbreviations

The following abbreviations are used in this manuscript:

CN	central neurocytoma
CNS	central nervous system
CT	computed tomography
EMA	epithelial membrane antigen
EVD	external ventricular drainage (EVD)
FET	F18-fluorethyltyrosin
GFAP	glial fibrillary acidic protein
GTR	gross total resection
Gy	gray
HE	hematoxylin–eosin staining
HPF	high-power field
IQR	interquartile ranges
MRI	magnetic resonance imaging
NeuN	Hexaribonucleotide Binding Protein-3
P53	tumor protein P53
PET	positron emission tomography
VP	ventricular–peritoneal
WHO	World Health Organization
